# Divergence in genital morphology may contribute to mechanical reproductive isolation in a millipede

**DOI:** 10.1002/ece3.466

**Published:** 2013-01-09

**Authors:** Janine M Wojcieszek, Leigh W Simmons

**Affiliations:** Centre for Evolutionary Biology, School of Animal Biology M092, University of Western Australia35 Stirling Highway, Crawley, Western Australia, 6009, Australia

**Keywords:** Lock-and-key, male genitalia, Millipede, population cross, sexual selection, species mate recognition

## Abstract

Genitalia appear to evolve rapidly and divergently in taxa with internal fertilization. The current consensus is that intense directional sexual selection drives the rapid evolution of genitalia. Recent research on the millipede *Antichiropus variabilis* suggests that the male genitalia are currently experiencing stabilizing selection – a pattern of selection expected for lock-and-key structures that enforce mate recognition and reproductive isolation. Here, we investigate how divergence in genital morphology affects reproductive compatibility among isolated populations of *A. variabilis*. Females from a focal population were mated first to a male from their own population and, second, to a male from one of two populations with divergent genital morphology. We observed variation in mating behavior that might indicate the emergence of precopulatory reproductive barriers: males from one divergent population took significantly longer to recognize females and exhibited mechanical difficulty in genital insertion. Moreover, we observed very low paternity success for extra-population males who were successful in copulating. Our data suggest that divergence in genital shape may be contributing to reproductive isolation, and incipient speciation among isolated populations of *A. variabilis*.

## Introduction

Species-rich taxa usually display significant divergence in traits related to reproduction (Darwin 1871). This pattern has led to much debate over whether sexual selection may underlie rapid divergence in sexual structures and, therefore, be an overlooked “engine of speciation” (West-Eberhard 1983; Panhuis et al. 2001; Turelli et al. 2001; Ritchie 2007; Sobel et al. 2010). Genitalia are arguably the most variable of all sexual structures, with striking differences apparent among taxa, including between closely related species (Eberhard 1985; Hosken and Stockley 2004). As with other sexual traits, it is thought that intense directional sexual selection drives the rapid evolution of divergent genitalia among isolated populations (Eberhard 1985, 2009, 2010; Arnqvist 1997, 1998; Sirot 2003; Hosken and Stockley 2004), potentially leading to an increased frequency of speciation (Arnqvist 1998; Panhuis et al. 2001). The classic hypothesis for genital evolution posits that genitalia function as “lock-and-key” structures, which are subject to stabilizing selection, enforcing mate recognition and species isolation (Dufour 1844; Shapiro and Porter 1989; Arnqvist 1997; Hosken and Stockley 2004). These processes need not be mutually exclusive. Species may go through periods of continuous directional evolution, punctuated by periods of stabilizing selection in which incipient species are reproductively isolated by the products of past directional selection. Stabilizing selection may be relatively more important to speciation in some taxa than others (McPeek et al. 2008; Hoskin and Higgie 2010). Understanding the relative contributions of different selection regimes to the evolution of diverse sexual structures, such as genitalia, is therefore important if we are to uncover the origins of reproductive isolation and the mechanisms underlying speciation.

Millipedes (Class Diplopoda) are a useful group for exploring the links between speciation and genital evolution, as species are almost exclusively identified and described using the morphology of the male genitalia (Sierwald and Bond 2007). Furthermore, millipedes generally display low vagility and so have an inherent tendency toward geographic isolation, which is likely to promote genetic divergence and ultimately speciation (Loomis and Schmitt 1971; Tanabe et al. 2001; Moir et al. 2009; Edward and Harvey 2010; Sota and Tanabe 2010). Indeed, the millipede genus *Antichiropus* is extremely diverse, with over 120 species occurring in south-western Western Australia, all with divergent male genital morphology (Harvey 2002). Recent research has investigated male genital evolution in one species – *Antichiropus variabilis*. Population genetic analyses, using both neutral microsatellite markers and mitochondrial DNA, have revealed strong genetic divergence among isolated populations of *A. variabilis* throughout the species range (Wojcieszek and Simmons 2012a). Male genital morphology has also undergone significant morphological divergence among these populations, but at a rate considerably slower than would be expected from genetic divergence at neutral loci. Such a pattern is unlikely to be due to genetic drift, and is characteristic of strong stabilizing selection, rather than directional selection, currently acting on male genital morphology within populations (Wojcieszek and Simmons 2012a). In addition, variation in genital morphology was found to affect male paternity success within one population of *A. variabilis* (Wojcieszek and Simmons 2011), and selection gradient analyses again revealed that male genitalia are currently experiencing stabilizing selection – the mode of selection expected for lock-and-key structures that function in mate recognition and species isolation (Wojcieszek and Simmons 2011). As only the specific “key” of a male can fit inside the “lock” of a conspecific female, morphological compatibility between the genitalia of males and females may be crucial for successful copulation and sperm transfer.

If the genitalia of *A. variabilis* are currently imposing reproductive isolation, we would expect that the observed divergence in genital morphology would reduce interpopulation mating capacity and/or competitive fertilization success, comprising a barrier to successful reproduction. Compared to pre and postzygotic reproductive barriers (Dobzhansky 1940; Howard and Gregory 1993; Coyne and Orr 2004), much less is known about reproductive barriers that exist during copulation, that is, barriers that operate after matings have begun, but before gametes make contact (Wade et al. 1994; Price 1997; Howard et al. 1998; Howard 1999; Eady 2001; Chang 2004; Fricke and Arnqvist 2004a; Jagadeeshan and Singh 2006). Barriers that prevent successful copulation may contribute to reproductive isolation (Eady 2001), and it is important to investigate how such barriers may emerge among taxa at different stages of evolutionary divergence (Price 1997; Dixon et al. 2003; Fricke and Arnqvist 2004a; Mendelson et al. 2007), including among incipient species. Divergent genital morphology may enforce mechanical barriers during copulation, preventing successful reproduction among populations of *A. variabilis*, and thus contribute to incipient speciation.

In this study, we mated females from a single focal population to two different males; first to a male from their own population, and secondly to a male from one of two populations that were geographically isolated from the focal population. We know the patterns of paternity expected from the Serpentine population when two males copulate with the same female (Wojcieszek and Simmons 2011); hence, we chose Serpentine as our focal population. We selected Gingin and Manjimup populations to source extra-population males because they displayed similar levels of genetic differentiation at microsatellite loci when compared with Serpentine (pairwise *F*_ST_ values between Serpentine and Manjimup, and between Serpentine and Gingin = 0.441 and 0.474, respectively; Wojcieszek and Simmons 2012a). Moreover, following a discriminant analysis of male genital morphology, the Serpentine and Gingin populations clustered closer in multivariate space than did the Serpentine and Manjimup populations, providing variation in the degree of genital divergence among extra-population males (Wojcieszek and Simmons 2012a). As second male sperm precedence was the outcome of double matings within the focal population (Wojcieszek and Simmons 2011), the current study assessed mating capacity and variation in second male sperm precedence for males from the two external populations. We predicted that extra-population males whose genitalia were divergent from within-population males would be less successful in achieving copulation and/or last male precedence in paternity, as their genitalia should be mechanically incompatible with those of the focal females. In addition, we predicted that Manjimup males would be less successful than Gingin males, as they displayed greater levels of genital divergence to the focal Serpentine population. Thus, we assess whether genital divergence constitutes a barrier to successful interpopulation reproduction among divergent populations of *A. variabilis*, as would be expected of a character involved in reproductive isolation and speciation.

## Methods

### Animal collection and housing

*Antichiropus variabilis* females were collected from Serpentine Falls National Park, Western Australia (32°22′01″S, 116°00′28″E) between 4 June and 6 July 2009. Males were collected from three localities in south-western Western Australia: (1) Serpentine Falls National Park between 11 June and 6 July 2009; (2) Boonanarring Nature Reserve, Gingin (31°10′28.7″S, 115°50′29.6″E) on 5 July 2009; and (3) Mersea Forest, North of Manjimup (34°05′07.6″S, 116°11′03.5″E) on 3 and 4 July 2009 (see Wojcieszek and Simmons 2012a for a map of locations). Animals were collected under license from the Department of Environment and Conservation, Western Australia (license numbers: SF006845 and CE002381). Millipedes were housed individually in transparent plastic containers under well-established laboratory conditions (Wojcieszek et al. 2011).

### Mating trials and behavioral observations

Mating trials were conducted between 7 and 14 July 2009, and involved single females (*N* = 34) being mated firstly to a male from their own population (Serpentine). We used a matched pairs design where a Serpentine male was mated to two different females. After at least 24 h (mean = 32.2 ± 3.0 h), females were then mated to a second male; within each matched pair, one female was mated to a Gingin male, whereas the other female was mated to a Manjimup male. Serpentine males were rested for at least 2 days before mating with the second female in the pair, such that any short-term sperm depletion following their initial mating could be avoided. During the mating trials, we recorded: (1) the time taken for a male to instigate mating after his antennae first touched a female; (2) the number of times a male's genitalia were withdrawn and reinserted into the female (during within-population matings, the male genitalia are only inserted once at the start of mating and remain within the female until mating ceases; Wojcieszek and Simmons 2011); and (3) the duration of copulation and the duration of the active phase (see Wojcieszek and Simmons 2011 for a description of mating in *A. variabilis*). Following matings, males were preserved in 100% ethanol, whereas females were put back into their individual containers. Females were monitored daily postmating and when hatched offspring were observed, females and offspring were preserved in 100% ethanol. One female died prior to egg-laying and the egg clutches from an additional four females failed to hatch. Thus, of the 34 females used in the experiment, offspring were obtained from 29 females, including 15 females where a Manjimup male was the second to mate, and 14 females where a Gingin male was the second to mate.

### Paternity assignment

Genomic DNA was extracted from all mothers (*N* = 29), potential fathers (*N* = 58), and a subset of between 20 and 24 offspring (except for one female where only 16 offspring were available). Details of molecular protocols, including DNA extraction, polymerase chain reaction (PCR) components, and PCR cycling conditions, are provided elsewhere (Wojcieszek and Simmons 2009, 2011). Two separate multiplex PCRs were run for each individual using primer pairs that amplified two and five polymorphic microsatellite loci, respectively (multiplex 1 and multiplex 3; Wojcieszek and Simmons 2009). In cases where further genetic information was required to assign offspring paternity definitively, an additional four loci were amplified (multiplex 2; Wojcieszek and Simmons 2009). Paternity was assigned manually using the exclusion approach (Wojcieszek and Simmons 2011). In instances where loci were mismatched, it was assumed that an unknown male had fathered offspring, suggesting that some females were not virgins at the time of collection. Paternity values assigned to each male corresponded to the true proportions of offspring sired. For example, if the first male sired 50% of the offspring genotyped, his paternity successes was assigned as 0.5; if the second male sired only an additional 10% of the offspring genotyped, his paternity success was assigned as 0.1. In this case, an unknown male sired the remaining 40% of offspring genotyped. Importantly, our approach did not remove the effect of unknown males, as this would have artificially inflated paternity success for some males.

### Variation in male genital morphology across populations

Male millipedes possess paired secondary genitalia known as gonopods (Hopkin and Read 1992). At the onset of mating, males will charge their gonopods with sperm that extrudes from their gonopores; males then insert both gonopods into the female genitalia. To assess variation in male genital morphology, the left gonopods of males were dissected, placed onto glass slides, and photographed using a Leica MZ6 binocular microscope and an AxioCam MRc5 camera (Zeiss, North Ryde, New South Wales, Australia). Geometric morphometric analyses (Zelditch et al. 2004) were used to quantify variation in the shape and size of the male genitalia. As in previous analyses with *A. variabilis*, 22 fixed and 13 sliding semilandmarks were digitized using the tpsDig2 v2.12 software (http://life.bio.sunysb.edu/morph; F. James Rohlf, Department of Ecology and Evolution, Stony Brook University, Stony Brook, NY). Tpsrelw v1.46 software (F. James Rohlf, see website above) was used to generate Relative Warps (RWs) and centroid sizes (see Wojcieszek and Simmons 2011 for a detailed description of the different landmarks used and the relative warps analysis performed). We conducted three separate geometric morphometric analyses: (1) including males from all three populations; (2) including only Manjimup males; and (3) including only Gingin males. Relative warp and centroid size variables obtained in the latter two morphometric analyses were used to determine whether variation in male genital morphology predicted paternity success for Manjimup and Gingin males in separate statistical tests (only RW scores 1–8 were used, as only these RWs each accounted for ≥1% of the variation in gonopod shape for both populations). We performed a discriminate function analysis (DA) in JMP® v7.0 (SAS Institute Inc.), using RW and centroid size variables from the analysis including males from all three populations. We obtained a single value for each individual Manjimup and Gingin male, which described their “morphological distance” to the Serpentine mean (distance between each individual's placement in multivariate trait space and the Serpentine centroid).

### Statistical analyses

We were interested in the paternity success and behavior of the last males to mate, as our within-population experiment revealed that the last male to mate fathered on average 70% ± 6% of a female's offspring (Wojcieszek and Simmons 2011). We also showed that within the Serpentine population, mating order (first or second male) had no significant effect on mating behavior (Wojcieszek and Simmons 2011). Thus, any differences in mating behavior observed in this study would be due to the population from which males were sourced, rather than their mating order. Because paternity success and behavioral variables were not normally distributed, nonparametric statistics were used. When comparing behavioral and paternity data of males, we used Wilcoxon signed-rank tests for matched pairs, reflecting our mating design. All statistical tests were completed using R v2.8.1 (R Development Core Team 2008), and results are presented as medians and interquartile ranges (IQR), unless otherwise stated.

## Results

### Mating behavior of males

**Figure 1:**
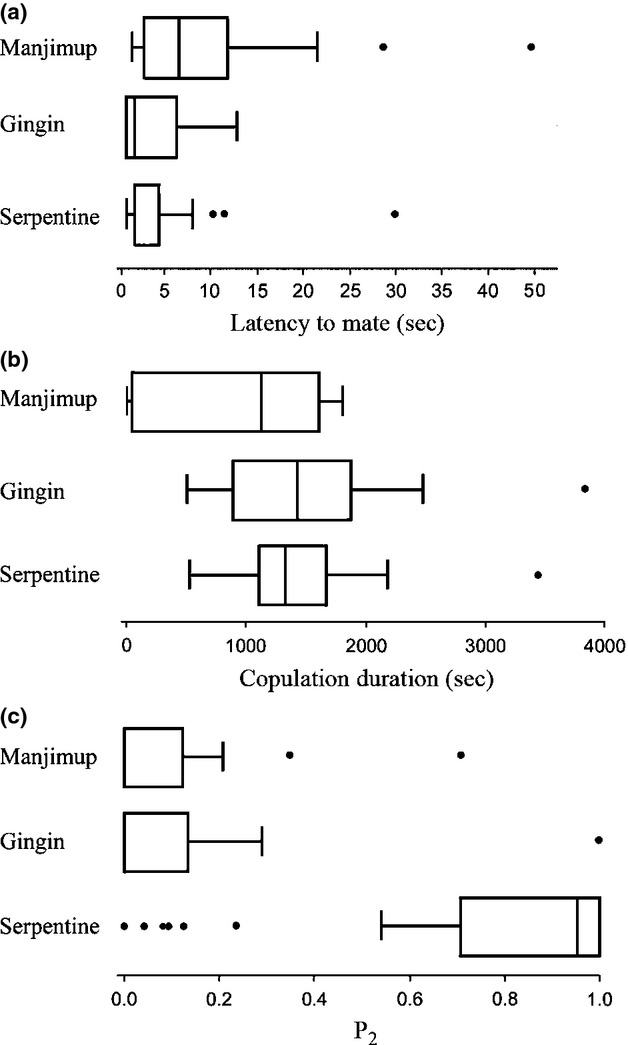
Differences in male behavior and paternity success among populations: (a) interpopulation differences in the time it took males to instigate mating once their antennae made contact with a Serpentine female; (b) interpopulation differences in the duration of copulation with Serpentine females; and (c) the distribution in paternity success of last males to mate with a Serpentine female. Note that Serpentine males were only mated in the first male role in the current experiment; we thus sourced P_2_ values for Serpentine males from our previous mating experiment (Wojcieszek and Simmons 2011).

A comparison of behavioral variables among populations is presented in Table 1. There was no difference in the time it took males from Serpentine and Gingin to initiate mating once a male's antennae touched a female (Wilcoxon signed-ranks test for matched pairs, *V* = 7, *P* = 0.27; Fig. 1a). It took Manjimup males significantly longer than Serpentine males to initiate mating with Serpentine females (Wilcoxon signed-ranks test for matched pairs, *V* = 65.5, *P* = 0.04; Fig. 1a). Only one Gingin male (7%) inserted his gonopods (secondary genitalia) more than once during mating. Six out of 15 Manjimup males (40%) withdrew and reinserted their gonopods into females between two and 14 times during mating. This was significantly different to the usual trend for within-population Serpentine matings, where males only inserted their gonopods once at the start of copulation (Wilcoxon signed-ranks test for matched pairs, *V* = 3, *P* = 0.04). The gonopods of some Manjimup males repeatedly “popped out” from the gonopores of females and often only one of the gonopods appeared to be inserted; these males often ceased matings abruptly. For example, one Manjimup male inserted his gonopods six times and after only 8 sec of genital contact, the male terminated the mating and walked away from the female. A further three Manjimup males only mated for between 44 sec and 4.1 min. There was no difference in copulation duration for Gingin males when compared to Serpentine males (Wilcoxon signed-ranks test for matched pairs, *V =* 48, *P =* 0.89; Fig. 1b). However, Manjimup males mated for a significantly shorter duration than did Serpentine males (Wilcoxon signed-ranks test for matched pairs, *V =* 20, *P =* 0.04; Fig. 1b).

**Table 1:** Mating behavior of males from three different populations when mated last to a Serpentine female. Data are presented as medians, with interquartile ranges in parentheses

	Serpentine males	Gingin males	Manjimup males
Latency to mate (sec)	2.0 (2.0–4.5)	2.0 (1.0–6.5)	7.0 (3.3–12.3)*
Active phase duration (sec)	10.0 (8.0–14.0)	11.0 (7.8–15.0)	10.0 (8.0–35.0)
Total copulation duration (min)	22.9 (18.6–27.7)	23.8 (15.0–31.3)	18.8 (0.7–26.9)*
Number of gonopod insertions	1.0 (1.0–1.0)	1.0 (1.0–1.0)	1.0 (1.0–4.5)*

* Manjimup male significantly different to Serpentine male (Wilcoxon signed rank test for matched pairs; *P* < 0.05).

### Paternity success

**Figure 2:**
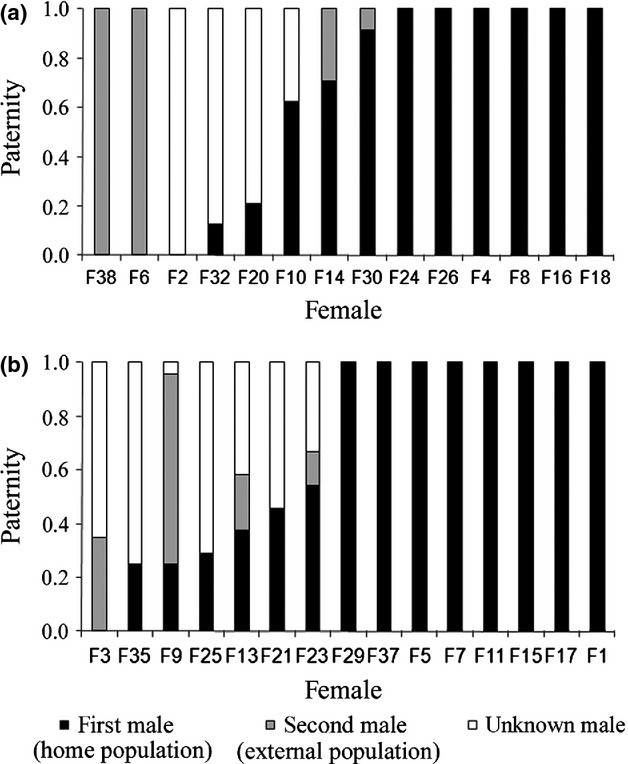
Stacked bar charts showing distribution of paternity success: (a) for females mated secondly to Gingin males, and (b) for females mated secondly to Manjimup males. At least 11 females had already mated in the field prior to collection, as evidenced by the offspring sired by “unknown” males.

The raw data for the paternity success for all males are shown in Fig. 2. There were several cases where multiple offspring were sired by unknown males, thus some females were not virgins at the time of collection (*N =* 11). There were two cases of complete last male precedence for a Gingin male, whereas there were no cases of complete last male precedence for any Manjimup males. Despite a slight trend for Gingin males to be more successful than Manjimup males in siring offspring (proportion of offspring sired: Gingin, median = 0, IQR = 0–0.135; Manjimup, median = 0, IQR = 0–0.125), there was no significant difference between the paternity success of males from the two populations (Wilcoxon signed-ranks test for matched pairs, *V* = 12, *P* = 0.80). A total of eight extra-population males were successful in obtaining paternity (four from each of the two populations). Of this subset of successful males, the Gingin males again obtained almost twice the paternity success, on average, as the Manjimup males (proportion of offspring sired: Gingin, median = 0.65, IQR = 0.14–1.0; Manjimup, median = 0.28, IQR = 0.15–0.62), but again the difference was not statistically significant (Mann–Whitney *U*-Test, *W* = 6, *P* = 0.66). Paternity success was significantly correlated with the total duration of copulation for Gingin males (*r* = 0.65, *P* = 0.01) and for Manjimup males (*r* = 0.51, *P* = 0.05), but not for Serpentine males (*r* = 0.01; *P* = 0.94). Paternity success was not correlated with any of the other behavioral variables measured, nor with the time elapsed between a female's first and second matings (all *P* values > 0.05). Serpentine males were only used in the first male role in the current experiment; we thus sourced P_2_ values (paternity success for second males to mate) for Serpentine males from our previous study (Wojcieszek and Simmons 2011). Males from both Manjimup and Gingin scored significantly lower paternity than did males from the female's home population of Serpentine when mating last to females (Manjimup v. Serpentine: Mann–Whitney *U-*Test, *W* = 38.5, *P* < 0.0001; Gingin v. Serpentine: Mann–Whitney *U-*Test, *W =* 80, *P* = <0.001; see Fig. 1c). Therefore, there was a trend for first male, or within-population, male precedence (mean P_1_ for Serpentine males in extra-population matings = 0.65 ± 0.07), rather than the expected trend of last male precedence (mean P_1_ for within-Serpentine matings = 0.23 ± 0.06; Fig. 1c; see: Wojcieszek and Simmons 2011).

### Paternity success and genital morphology

**Figure 3:**
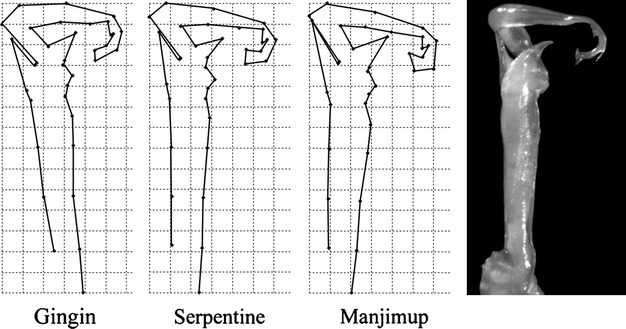
A comparison of consensus shapes for gonopods from males from each of the three populations. Serpentine and Gingin males have similar genital morphology, whereas Manjimup males have more divergent genitalia, especially in the two medial projections. On the right, a photograph showing a dissected left gonopod from a Serpentine male.

**Figure 4:**
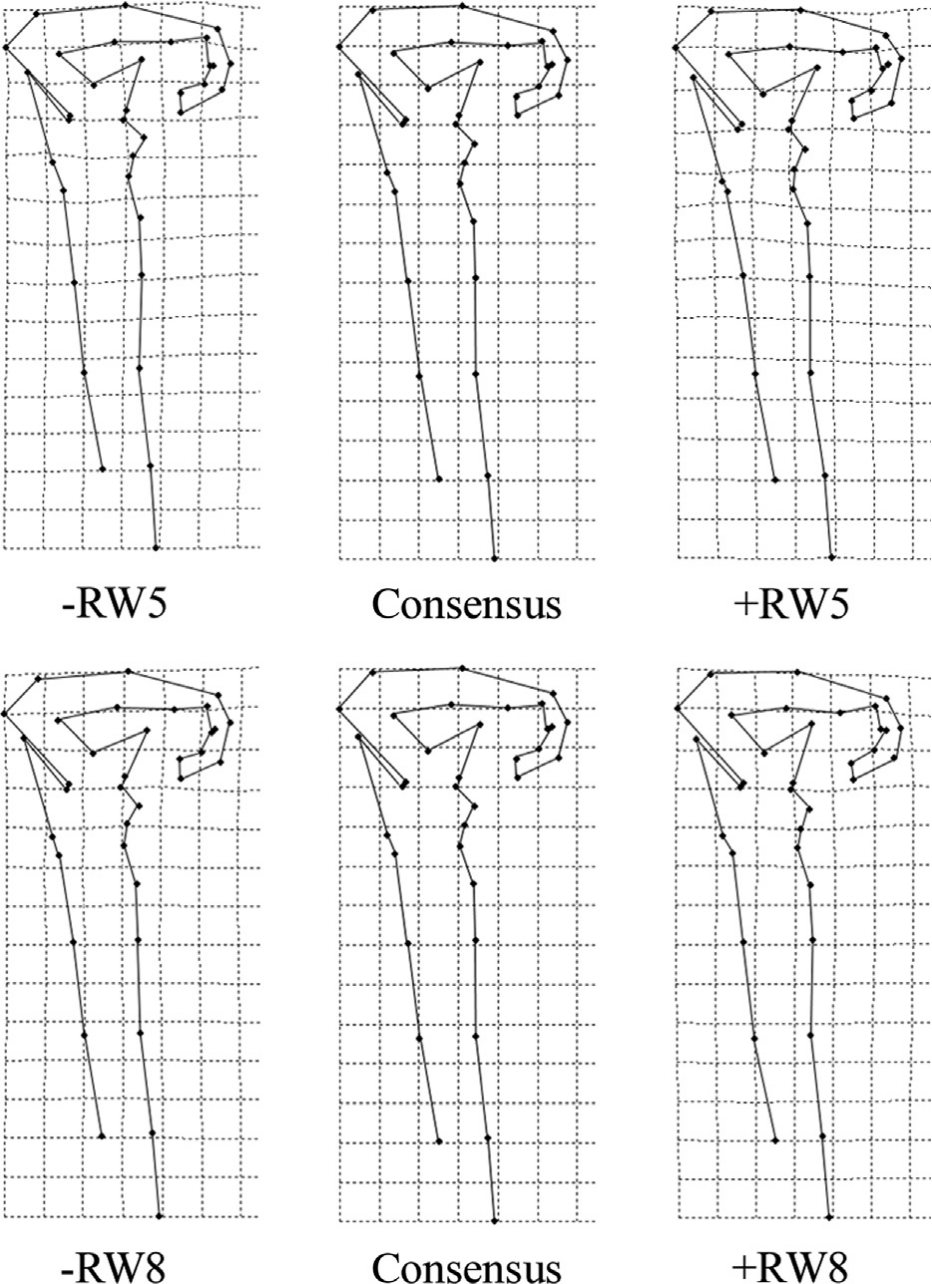
Thin plate splines showing extremely subtle shape variation as described by the two relative warps (RW5 and RW8) that influenced the paternity success of Gingin males. Consensus shapes, following geometric morphometric analysis, are shown in the center images, alongside a comparison of how extreme negative values (left images) and extreme positive values (right images) of relative warps cause extremely subtle conformational changes in shape, as highlighted by the arrows. Landmark positions have been “joined” using straight lines to show the shapes clearly.

Consensus shapes for male genitalia for each of the three populations are shown in Fig. 3. There was no relationship between an individual male's paternity success and his morphological distance to the Serpentine centroid: for Gingin males (*r* = 0.10, *P* = 0.70); for Manjimup males (*r* = 0.39, *P* = 0.15). We also tested whether gonopod morphology predicted paternity success using morphometric variables obtained in the separate population-specific relative warps analyses. For the Manjimup males, paternity success was not correlated with gonopod size (*r* = 0.10, *P* = 0.73), or gonopod morphology (*r* and *P* values for each RW ranged from 0 to 0.47 and from 0.07 to 0.97, respectively). There was no relationship between paternity and gonopod size for Gingin males (*r* = 0.0; *P* = 0.84). However, there was a significant correlation between paternity success and genital shape described by RW5 (*r* = 0.60, *P* = 0.02) and RW8 (*r* = 0.62, *P* = 0.02) for the males from Gingin. Variation in gonopod shape of Gingin males described by these two RWs represents very subtle deviations in the shape of the distal gonopod “swirl” and the medial projections; this is shown in Fig. 4. Relative warp 5 accounted for 5.2% of the variation, and RW8 accounted for 2.9% of the variation in gonopod shape within Gingin males.

## Discussion

This study has explored how divergence in male genitalia may contribute to reproductive isolation and incipient speciation among isolated populations of the millipede *A. variabilis*. We found that when extra-population males mated last to Serpentine females, although copulation was achieved, it was associated with obvious mechanical difficulties and significant variation in copulation duration. Importantly, we observed that extra-population males were capable of fathering offspring, but that this was uncommon. We therefore observed a clear breakdown of the known pattern of second male sperm precedence, instead observing first male, and thus within-population, sperm precedence. We cannot rule out the possibility that mortality of hybrid embryos may have contributed in part to the poor success of extra-population males (Fricke and Arnqvist 2004a). However, given that some extra-population males were successful in siring offspring, including two cases of complete sperm precedence, postzygotic barriers to reproduction are certainly not complete. More importantly, the fact that extra-population males experienced difficulties in copulation, and that among those that did sire offspring, genital morphology, and the duration of copulation both influenced the proportion of offspring sired, suggests that reproductive barriers operating during mating and copulation contributed to the observed patterns of paternity.

Our experiment has confirmed that divergence in genital morphology can contribute to mechanical reproductive isolation among incipient species (Coyne and Orr 2004). Both Manjimup and Gingin males experienced difficulties in copulating and achieved low paternity with Serpentine females. We had expected to see greater genital incompatibility between Manjimup and Serpentine pairings, as Manjimup males have a greater degree of morphological divergence from Serpentine males than do Gingin males (Wojcieszek and Simmons 2012a). Indeed, we did find qualitative and quantitative differences between Manjimup and Gingin males. Manjimup males withdrew their gonopods more often during copulation and achieved shorter copulations than did Gingin males. Manjimup males sired fewer offspring than Gingin males, although this difference was not statistically significant, possibly due to the smaller than anticipated sample size. We detected a correlation between copulation duration and paternity success for Gingin and, to a lesser extent, Manjimup males, whereas there was no such correlation for within-population Serpentine males. Finally, we also observed that some of the variation in gonopod shape, but not size, was associated with paternity success for Gingin males, as found for within-population Serpentine males (Wojcieszek and Simmons 2011). In contrast, paternity remained low, independent of genital morphology for Manjimup males. Although we are yet to investigate the patterns of variation in female genital structures in detail, micro-CT scanning of *A. variabilis* genitalia *in copula* has revealed how shape is important for optimal genital coupling (Wojcieszek and Simmons 2012b), and it appears that even slight divergence in genital shape, as seen in Gingin males, may lead to a breakdown in the mechanisms that usually take place during copulation, greatly reducing reproductive compatibility. The even greater divergence in genital shape of Manjimup males appeared to generate a poor fit with serpentine female genitalia resulting in Manjimup males experiencing considerable difficulty in genital insertion. Our data therefore suggest the emergence of mechanical reproductive barriers among divergent populations of this species, with significant genital divergence leading to detectable morphological incompatibilities among the populations surveyed.

Our study has shown that divergence in genital shape prevented successful genital coupling among populations of the same species. The findings correspond with earlier research suggesting that paternity success in *A. variabilis* is partially dependent on a “lock-and-key” fit between male and female genitalia (Wojcieszek and Simmons 2011, 2012a,b). Genitalia that function as lock-and-key structures are rare in nature (Shapiro and Porter 1989; Coyne and Orr 2004), most likely because the majority of taxa usually achieve species mate recognition prior to genital contact (Rentz 1972; Wojcieszek and Simmons 2011). Indeed, we also found evidence for premating incompatibilities among our extra-population males. Compared to Gingin males, Manjimup males took significantly longer to “recognise” and mate with Serpentine females following antennal contact. Cuticular chemicals and genital morphology may comprise a two-tiered species recognition system in *A. variabilis* millipedes (and possibly other millipedes; see Tanabe and Sota 2008). Although Manjimup males took significantly longer to recognize females following antennal contact, they did eventually attempt matings. Mechanical barriers may therefore be evolving faster than premating barriers among the populations investigated in this study (see also Sota and Kubota 1998; Dixon et al. 2003; Fricke and Arnqvist 2004b; Tanabe and Sota 2008). As premating isolation was incomplete among populations, the low incidence of paternity success for extra-population males provides evidence that divergent genital morphology may function as a “back-up” isolating mechanism in *A. variabilis* (Eberhard 1985; Mutanen et al. 2006; Tanabe and Sota 2008; Wojcieszek and Simmons 2011).

Species-rich taxa usually display significant divergence in traits related to reproduction (Darwin 1871). This has led to much debate over whether sexual selection may underlie rapid divergence in sexual structures and thus drive speciation (West-Eberhard 1983; Panhuis et al. 2001; Turelli et al. 2001; Ritchie 2007; Sobel et al. 2010). Indeed, comparative analyses of some birds (Barraclough et al. 1995; Mitra et al. 1996), insects (Ringo 1977; Arnqvist et al. 2000), and fish (Mank 2007) have suggested a link between taxonomic diversity and the intensity of sexual selection. However, further comparative studies of birds (Morrow et al. 2003), mammals, butterflies, and spiders (Gage et al. 2002), and of Mexican Goodeid fish (Ritchie et al. 2005), have all failed to find conclusive evidence that sexual selection promotes speciation. The potential for sexual selection to promote speciation therefore remains contentious (West-Eberhard 1983; Questiau 1999; Panhuis et al. 2001; Turelli et al. 2001; Arnqvist and Rowe 2005; Ritchie 2007; Hoskin and Higgie 2010; Sobel et al. 2010).

Divergence in sexual traits may occur for reasons other than directional sexual selection (Ritchie et al. 2007; Hoskin and Higgie 2010). The classic model of speciation posits that reproductive isolation, and thus the initial divergence in sexual traits, originates as an incidental by-product of population-specific adaptation and divergence following allopatric separation (Mayr 1942; Dobzhansky 1951; Schluter 2001; Coyne and Orr 2004). Our study has shown that morphological divergence in genitalia among populations or species can lead to low fertilization success, due to mechanical difficulties with copulation and/or poor success during postcopulatory sperm competition (Parker 1970) and/or cryptic female choice (Eberhard 1996). Nonetheless, premating isolation appears incomplete among the populations investigated in this study, thus, if allopatric populations were reunited in sympatry, sexual interactions could occur among incipient species with divergent genitalia. Some *A. variabilis* populations are currently sympatric with other *Antichiropus* species, whereby interactions with individuals from closely related species might lead to even greater levels of mate discrimination and thus greater genital divergence for these populations (Hoskin and Higgie 2010). Under these conditions, it is possible for reinforcement to operate, whereby hybrid matings may result in physical damage due to extensive mechanical incompatibilities (Sota and Kubota 1998; Usami et al. 2006; Sota and Tanabe 2010). Individuals that attempt hybrid matings could therefore suffer reduced lifetime fitness (Sota and Kubota 1998), and individuals better able to identify suitable mates, or individuals that are prevented from mating due to overwhelming morphological incompatibilities, may have comparatively higher lifetime fitness (Sota and Kubota 1998; Higgie and Blows 2008; Kameda et al. 2009). In time, reproductive character displacement of genital morphology is expected to evolve to reduce the occurrence of costly matings and hybridization (Sota and Kubota 1998; Kawano 2002, 2004; McPeek et al. 2008; Kameda et al. 2009). Premating reproductive isolation may also evolve to prevent mating following secondary contact with a divergent lineage (Schluter 2001). Therefore, both pre and postcopulatory mechanisms, such as cuticular chemicals and genitalia, could reinforce divergence and lead to an increased likelihood of speciation (Howard and Gregory 1993; Questiau 1999; Schluter 2001; Turelli et al. 2001; Marshall et al. 2002; Hoskin et al. 2005; Kameda et al. 2009; Hoskin and Higgie 2010). As traits targeted by sexual selection are often also involved in species mate recognition, divergence in sexual structures may influence mate choice, potentially altering patterns of gene flow, and promoting reproductive isolation and subsequent speciation in many taxa (Templeton 1979; Paterson 1993; Questiau 1999; Panhuis et al. 2001; Higgie and Blows 2008; Hoskin and Higgie 2010).

In conclusion, the genitalia of *A. variabilis* appear to function as lock-and-key like structures, and our study suggests that genital divergence may be contributing to mechanical reproductive isolation among populations of *A. variabilis* that appear to be undergoing incipient speciation. While directional sexual selection can clearly play a important role in the evolutionary divergence of male genital morphology, we suggest that processes of mate recognition and species isolation may also contribute to the evolution of divergent genital morphologies in *Antichiropus* and possibly other speciose groups, especially those taxa with low vagility.
